# Isolation and Characterization of a Rhizobacterial Antagonist of Root-Knot Nematodes

**DOI:** 10.1371/journal.pone.0085988

**Published:** 2014-01-21

**Authors:** Lihui Wei, Ying Shao, Jingwang Wan, Hui Feng, Hua Zhu, Huiwen Huang, Yijun Zhou

**Affiliations:** Institute of Plant Protection, Jiangsu Academy of Agricultural Sciences, Nanjing, People's Republic of China; Graz University of Technology (TU Graz), Austria

## Abstract

The rhizobacterial strain Jdm2 was isolated from the rhizosphere of the traditional Chinese medicinal herb *Trichosanthes kirilowii* in Jiangsu province, China, and was identified as *Bacillus subtilis*. Exposure of cell-free filtrate of the strain to the root-knot nematode *Meloidogyne incognita* under *in vitro* conditions caused substantial mortality of the second stage juvenile (J2) and significantly reduced egg hatchability. A greenhouse trial demonstrated that 56 days after treatment with Jdm2, the number of galls associated with *M. incognita* infection in the tomato (*Solanum lycopersicum*) roots was significantly reduced compared to controls, and the disease severity of infected plants was lower in treated plants (36%) compared to water control (75%). Consistently, in the field trial, the biocontrol efficacy of Jdm2 reached 69%, 51% and 48% after 30, 60 and 90 days post-transplantation, respectively. As indicated by PCR-DGGE analysis, inoculation with Jdm2 strain had an effect on the bacterial community of the tomato rhizosphere at the first stage, but was not able to imperil the bacterial community stability for long time. The novel bacterial strain Jdm2 enhances plant growth and inhibits nematode activity, and has the potential to be a safe and effective microbial pesticide.

## Introduction

Plant-parasitic nematodes are important pathogens in agricultural production, which attack the roots of plants and cause root dysfunction, reducing rooting volume and the foraging and utilization efficiency of water and nutrients [1], [2]. Some species of the genus *Meloidogyne* are obligate parasites that live in plant roots among a wide range of crops, particularly vegetables, causing dramatic yield reductions, mainly in tropical and sub-tropical agricultural areas. The disease causes reductions in yield most commonly by 15-25%, however in severe cases it can be up to 75% [3], [4]. Worldwide estimated losses per year caused by root-knot nematodes are over $100 billion [5].

The application of nematicides can alleviate disease severity, but it's harmful to animals and humans, and may cause environmental pollution, leading to a growing interest in finding safe substitutes for the control of plant-parasitic nematodes. Alternatives of chemical approaches for plant-parasitic nematode control include crop rotation and the use of biological control agents [6].

Accordingly, thousands of microbial strains have been screened, and many have been found to be antagonistic to plant-parasitic nematodes [7]–[10]. However, few biocontrol products are currently commercially available and it is necessary to find different strains of antagonistic bacteria for controlling plant-parasitic nematodes more efficiently [11].

The aims of the present study were: (1) to characterise the rhizobacterial strain Jdm2 isolated from the traditional Chinese medicinal herb *Trichosanthes kirilowii* in detail, and evaluate its biocontrol efficacy on *Meloidogyne incognita*, and (2) to assess the effects of inoculation of the strain Jdm2 on the bacterial communities in the rhizosphere of tomato plants by denaturing gradient gel electrophoresis of 16S rRNA gene fragments amplified from total community DNA under field conditions.

## Results

### Effect of the antagonist on J2 mortality and egg hatchability of M. incognita

**Figure 1:**
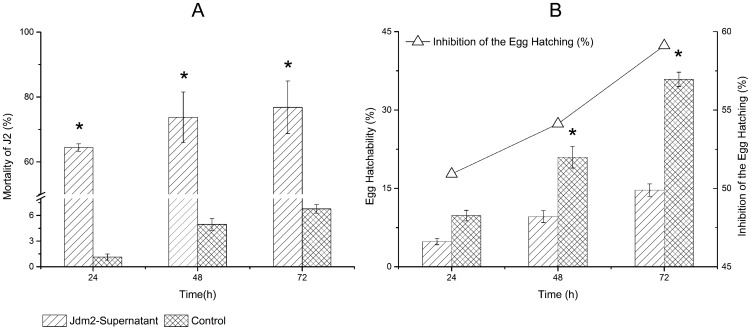
Effect of the strain Jdm2 on J2 mortality and egg hatching of *M. incognita*. About 100 J2 (A) or 1000 eggs (B) of *M. incognita* were soaked in antagonist supernatant with a water control. Values are mean±SEM (Data is mean of three replicates). “*” above the bars indicate significant differences at 5% probability level.

The cell-free culture filtrate of the antagonistic bacterial strain Jdm2 demonstrated nematicidal effects, killing the second stage juvenile (J2) of *M. incognita*. The increase in the mortality of juveniles positively correlated with the length of exposure. The relative mortality rates of *M. incognita* J2 caused by the antagonist reached 64%, 74% and 77% at 24 h, 48 h and 72 h post-inoculation, respectively (Figure 1A). The culture filtrate of the strain also exhibited potent effects on the inhibition of egg hatching. When compared to the control, the culture filtrate dramatically decreased the egg hatchability after 48 h post-inoculation (Figure 1B).

### Identification of the Jdm2 strain

The detailed morphological and physiological characteristics were summarized in Table 1. Based on these characteristics, Jdm2 belongs to the *B. subtilis* group. Furthermore, the 16S rRNA gene sequence of the antagonist Jdm2 had 100% similarity to *B. subtilis* (EU771076). Thus the phylogenetic study confirms that Jdm2 belongs to *B. subtilis*.

**Table 1:** Morphological and Physiological Characteristics of Strain Jdm2.

Characteristics	Reaction	Characteristics	Reactions
Endospore enlargement	−	Using of carbohydrate to produce acid	
Endospore round shape	−	Glucose	+
Parasporal crystal formations	−	Xylose	+
Catalase activity	+	L-Arabinose	+
Oxidase activity	+	Manitol	+
Anaerobic growth	−	Lactin	−
Voges-Proskauer test	+	Citrate test	+
Voges-Proskauer test<pH 6	+	Growth at 50°C	+
Voges-Proskauer test>pH 7	−	Growth at pH 5.7	+
Methyl red test	+	Growth at 7% NaCl	_
Nitrate deoxidize	+	Hydrolysis of starch	+
Gelatin liquefaction	+	Decomposition of casein	+

+, positive reaction; -, negative reaction.

### Biocontrol efficacy of Jdm2 against M. incognita and the biomass changes of tomato plants in greenhouse tests

The number of galls per tomato root system was counted 56 days after inoculation with the nematodes. Disease severity corresponding to the treatment of antagonist Jdm2 against *M. incognita* was 36%, which was significantly lower than that of the control, with a disease severity of 75%, indicated that the strain Jdm2 used as soil drench was able to decrease the galls of *M. incognita* (Table 2). At the same time, the effect on the plant growth of the inoculation of *Bacillus subtilis* Jdm2 was investigated. Shoot length, fresh and dry weight of tomato plants were significantly increased, which showed 26%, 15% and 35% higher biomass after the inoculation of Jdm2, when compared to un-inoculated controls. Based on this data, namely the ability to decrease disease severity and increase plant biomass, Jdm2 was selected for use as the biocontrol agent in the field experiment for controlling root-knot nematodes.

**Table 2:** Biocontrol efficacy of Jdm2 against *M. incognita* and their biomass changes of tomato plants in the greenhouse tests.

	Root		Shoot					
Treatments^1^	Disease severity/%	Biocontrol Efficacy/%	Length/cm	Increase of Length/%	Fresh weight/g	Increase of Fresh weight/%	Dry weight/g	Increase of Fresh weight/%
Jdm2	36.43±15.98^2^ b^3^	51.43	81.39±6.66 a	26.15	52.81±4.65 a	15.24	5.97±1.22 a	35.52
Control	75.00±10.92 a	–	64.51±9.85 b	–	45.82±6.26 b	–	4.41±1.42 b	–

1 Plant inoculated with antagonist suspensions at a concentration of 10^9^ CFU/mL with a water control. Each plant was inoculated with 10 egg masses.

2 Standard deviation of means.

3 Numbers followed by different letters in the same columns are significantly different from each other at 5% probability level. Data is mean of three replicates.

### Field tests of antagonistic strain Jdm2

In field tests, Jdm2 exhibited a multidimensional ability of controlling the disease caused by *M. incognita* as well as promote plant growth (Table 3). The biocontrol efficacy of the antagonistic strain was 69% at 30 d post-inoculation, 51% at 60 d post-inoculation and 48% at 90 d post-inoculation. The biocontrol efficacy of the group treated with avermectins declined over time, as we observed 73% efficacy at 30 days post-inoculation, 34% at 60 days post-inoculation and 26% at 90 days post-inoculation. We also inoculated a combination of Jdm2 and avermectins into the tomato rhizosphere, but the results didn't show much difference from the Jdm2-only group.

**Table 3:** Field tests of antagonistic strains Jdm2.

	Biocontrol Efficacies							
	30d		60d		90d		Yield increase	
Treatments^1^	Disease severity/%	Biocontrol Efficacy/%	Disease severity/%	Biocontrol Efficacy/%	Disease severity/%	Biocontrol Efficacy/%	Yield/kg	Increase of Yield/%
Jdm2	13.00±1.70^2^ b^3^	69.05	35.83±5.40 b	51.13	42.00±4.07 c	48.15	170.62±7.54 a	31.14
Avermectins	11.50±0.95 b	72.62	48.33±4.68 b	34.09	60.00±4.88 b	25.93	148.39±2.01 b	14.06
Control	42.00±4.78 a	–	73.33±5.57 a	–	81.00±3.44 a	–	130.09±5.88 c	–

1 Plants were treated with antagonist suspensions at a concentration of 10^5^ CFU/mL (500 mL for one seedling) or with the insecticide Avermectins, while control was a treatment with water.

2 Standard deviation of means.

3 Numbers followed by different letters in the same columns are significantly different from each other at 5% probability level. Data is mean of four replicates.

Fruit yields were also tested in the field experiment, in order to evaluate the potential of the bacterial agent as a pesticide. Our data indicated that Jdm2 increased the yield of tomatoes by 31%, while avermectins only increased yield by 14%.

### Cultivation-independent monitoring of Jdm2 effects on the bacterial community

**Figure 2:**
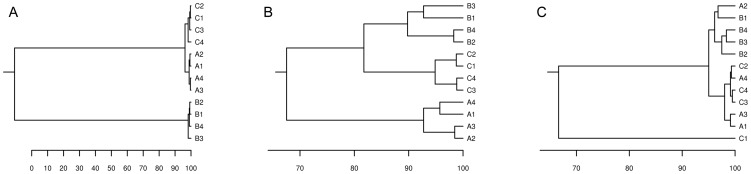
Dendrograms based on similarity matrices determined from the clusters analysis using the Pearson coefficient indices by means of unweighted pair group method using arithmetic averages (UPGMA) at 15, 30, and 60 days (A, B and C) post-inoculation. Scaling stands for similarity. ‘‘B1 –B4’’ was on behalf of the treatments amended with Jdm2, ‘‘A1–A4’’ was on behalf of the controls amended with Avermectins, and ‘‘C1–C4’’ was on behalf of the controls amended with water.

The bacterial community composition in tomato rhizospheres from different treatments was compared by DGGE analysis. Additionally, in order to determine the shifts in the relative abundance and diversity of bacterial ribotypes in the tomato rhizosphere as a consequence of inoculation with Jdm2, the DGGE fingerprinting was analyzed over time (Figure S1). Clustering and statistical analyses of bacterial community fingerprints were performed based on Pearson correlation indices. The result suggested that the influence of Jdm2 treatments on bacterial community in tomato rhizosphere decreased over time. At 15 days post-inoculation, the bacterial community composition of Jdm2-amended soils was dramatically different (d-value >105) from those for control (Figure 2A). At 30 days, the difference between samples Jdm2 treated and the non-treated control was less (d-value = 24.4) (Figure 2B). While at 60 days, only minor difference (d-value = 0.9) was observed between treatment of Jdm2 and control (Figure 2C).

To investigate the effects of inoculation on the *Bacillus* and *Pseudomonas* communities, they were analyzed by DGGE analysis. Highly similar *Bacillus* and *Pseudomonas* patterns in the rhizosphere of both the inoculated and control plants indicated that inoculation with Jdm2 had no effect on the *Bacillus* and *Pseudomonas* community composition in the rhizosphere (Figure S2).

## Discussion

It is of great interest to identify effective biocontrol agents against plant parasitic nematodes. Several studies have described the successful biocontrol of plant parasitic nematodes using bacteria [6]. The majority of *in vitro* screening strategies leverage the antagonistic strains' ability to increase J2 mortality or to suppress egg hatching [10]. Numerous studies have reported that bacterial culture filtrates also show nematicidal activity *in vitro* [12]-[18]. In this paper, the results indicate that the culture filtrates of the *B. subtilis* strain Jdm2 significantly increased J2 mortality and inhibited the hatching rate of the eggs compared with controls. The further study found that Jdm2 showed strong protease, chitinase and siderophore activity (data not shown). An increasing number of researchers have suggested that antibiotics, extracellular enzymes and other toxic compounds present in metabolites of rhizobateria are probably involved in the increase of J2 mortality and the inhibition of egg hatching. Siddiqi and Shaukat [15] reported that production of the metabolite 2,4-diacetylphloroglucinol (2,4-DAPG) by *Pseudomonas fluorescens* strain CHA0 inhibited egg hatching and induced mortality in J2 root-knot nematodes. Wang et al. [19] reported that an extracellular alkaline protease (Hasp) had effects of killing J2 of *Heterodera glycine* and degrading nematode cuticle proteins. Xia et al. [20] reported that the culture supernatant of *B. subtilis* strains OKB105 caused J2 of *M. incognita* a higher mortality than the strain OKB105 with a mutated *purL* gene.

In this study, we compared the biocontrol efficacy of Jdm2 with the avermectins. Avermectins offer an outstanding alternative to any of the available synthetic nematicides because of their novel mode of action, high potency and specific physico-chemical propertied and rapid degradation in soil and poor leaching potential [21]. We did not observe any significant differences on day 30 and day 60 after inoculating plants with either antagonist or insecticide. However, after 90 days, Jdm2 displayed an approximately two-fold increase in biocontrol efficacy compared to avermectins (48% compared to 26%). We assumed that combining a bacterial agent and an insecticide may enhance the capacity of controlling the disease, so we inoculated the tomato rhizosphere with a combination of Jdm2 and avermectins and found there was no big difference between Jdm2 and Jdm2+avermectin treatment, which was opposite to our hypothesis (data not shown).

Under both greenhouse and field conditions, Jdm2 displayed a potent efficacy that both reduced the severity of diseases caused by *M. incognita* and promoted growth in tomato seedlings. Although the mechanism of action of Jdm2 was not elucidated in these experiments, it may be hypothesized that nematode reduction may be attributed to a direct effect of Jdm2 metabolites that inhibit egg hatch and kill J2 of *M. incognita*. At the same time, Jdm2 around the roots was likely to induce plants systemic resistance and enhance host defenses, which restrained nematodes invasion and subsequent infection. Siddiqi and Shaukat [15] reported that *P. fluorescens* CHA0 could induce systemic resistance to root-knot nematode in tomato pants. BioYield, a commercial product that contains spores of the *B. subtilis* strain GB03 and *B. amyloliquefaciens* strain GB99 on a chitosan carrier, showed a property of promoting growth in tomato seedlings and reduce the severity of diseases caused by several pathogens [22]. Reduction of soil-borne pathogens was related to antibiotic production by GB03, while the promotion of indigenous soil predators and antagonists to root-knot nematodes was attributed to chitosan, and the elicitation of induced systemic resistance was attributed to *B. amyloliquefaciens* [23]. The growth promotion mechanism is dependent on the substances released from bacteria or nutrients absorption efficiency [24].

From the view point of biological invasion, the inoculation of a microorganism may disturb the original ecological balance of the soil microbial community [25], [26]. Some researchers also found that there were only transient effects on soil microbial communities following inoculation with biocontrol agents such as *P. fluorescens* [27], [28], *Streptomyces melanosporofaciens* [29] and *Corynebacterium glutamicum* [30]. Our results demonstrate that inoculation of Jdm2 had dramatically effects on the soil bacterial community at first, but the bacterial community recovered as the control 30 days later. In addition, the effects on the *Pseudomonas* and *Bacillus* community compositions were analyzed individually as these taxa often comprise populations with antagonistic activity and might not belong to the abundant populations in the rhizosphere. These results demonstrate that the Jdm2 strain not only possesses biocontrol potential, but also does not influence the soil bacterial community. Therefore, the antagonistic strain Jdm2 can be used as a biocontrol agent for root-knot nematodes.

## Materials and Methods

### Ethics Statement

No specific permits were required for the described field studies.

### Antagonistic bacterial strain and nematode preparation

An antagonistic bacterial strain with strong protease, chitinase and siderophore activities was selected, which displayed antagonistic activity toward bacterial and fungal plant pathogens, such as *Ralstonia solanacearum*, *Phytophthora capsici* and *Botrytis cinerea* (unpublished data).

The antagonistic strain was cultured on modified PDA medium (200 g potato, 10 g glucose, and 10.0 g agar per liter, at pH 7.2) at 30°C for 1-2 days before *in vitro* experiments.

In this study, the avermectins B1 (North China Pharmaceutical Company Aino Co., Ltd), of which the water solubility is approximately 9 ppm, was used as control 2.

*M. incognita* was originally isolated from the root of the Chinese medicinal herb *Trichosanthes kirilowii* [31]. Egg masses of *M. incognita* were collected using sterile forceps from the roots of heavily infected tomatoes. The egg masses were washed three times with sterile distilled water and then placed in Petri dishes containing just enough sterile water to keep the eggs wet. The hatched juveniles were harvested from the Petri dishes every 24 h to be used for inoculation, and fresh water was added to the dishes to prevent the eggs from drying.

### Effect of the antagonistic strain on J2 mortality and egg hatching of M. incognita

Bacterial strains to be tested were cultured in conical flasks and modified PD broth (containing 200 g potato and 10 g glucose per liter, at pH 7.2), and incubated at 30°C on a shaker (180 rpm) for 48 h. The bacterial culture (100 mL) was centrifuged at 5000 g for 15 min at 4°C, and the supernatant was saved for the following experiments.

To determine the effects of extracellular metabolites of the antagonists on J2 mortality, 2 mL of the culture supernatant of strain Jdm2 was transferred into a Petri dish with 1 mL of suspension containing 100 surface-sterilized J2 juveniles. The dishes were incubated at 25°C for 72 h, and the number of dead J2 was counted. The juveniles were considered dead when they did not move on probing with a fine needle [32]. In the control, the culture supernatant was replaced with the same volume of sterile modified PD broth. J2 mortality was calculated according to the formula: JM (%)  =  (T/C) × 100, where JM represents the mortality of J2, T represents the number of dead J2, and C the total number of J2 used in test.

To assess the effects of extracellular bacterial metabolites on *M. incognita* egg hatching, 2 mL of the culture supernatants were incubated in a Petri dish with 1 mL of egg suspension containing about 1000 surface-sterilized eggs. The egg suspension was prepared according to McClure et al. [33]. As a control, 1 mL of egg suspension was incubated with 2 mL of modified PD broth. The dishes were incubated at 25°C for 72 h. The inhibition of the egg hatching rate was calculated according to the formula: I (%)  =  (C-T)/C × 100, where I represents the inhibition of the egg hatching, T represents the eggs hatchability in the treatment groups, and C represents the eggs hatchability in the control group.

### Biocontrol efficacy of the antagonist strain against M. incognita in greenhouse tests

Tomato seeds (*Solanum lycopersicum*, cultivar ‘Shanghai Cooperation 903’ provided by the Chinese Academy of Agricultural Sciences) were surface-sterilized with 2% sodium hypochlorite for 2 min. The seeds were then washed thoroughly with sterilized water and planted in sterilized soil in pots, and maintained in a greenhouse at 25°C for 4 weeks. Seedlings were transplanted to new 10 cm diameter pots containing ‘Ready Mix’ (a fertilizer from Huai-an Chai-Mi-He Ready Mix and Fertilizer Company, Jiangsu, China), and were maintained at 25°C in a greenhouse with a relative humidity of 40% and a 12 h/12 h photoperiod for 2 days.

The individual seedlings in each pot were first inoculated with an antagonist by carefully pouring 40 mL of bacterial suspension (about 10^9^ CFU/mL) into the rhizosphere of each plant. The inoculated pots were maintained under the same conditions as described above for 2 additional days. Each pot was inoculated with 10 mL of a nematode suspension containing 600 freshly hatched J2. As a control, an equal amount of sterilized water, instead of antagonist suspension, was added to the pots.

Each treatment consisted of three replicates, and each replicate had 24 plants. All pots were arranged in a randomized block design. The number of galls per root system was counted 56 days after inoculation with the nematodes. The gall indices were confirmed using the 0-11 grade by Bridge and Page's method [34], Disease severity and biocontrol efficacy were calculated following the method of Xue et al., [11]:

Disease severity (%)  =  [Σ (The number of diseased plants in this index × Disease index)/(Total number of plants investigated × The highest disease index)] × 100.

Biocontrol efficacy (%)  =  [(Disease severity of control – Disease severity of antagonist-treated group)/Disease severity of control] × 100.

For surveying promotion of plant growth, tomato seeds and seedlings were treated as described above, except that they were not inoculated with *M. incognita*. Fifty-six days after treatment with the strain, the length, fresh and dry weights of the entire plants were measured, and the increase in biomass was calculated using the following formula: *BI*(%) = (*T*−*C*)/*C*×100

Where BI represents the increase of the biomass (length, fresh/dry weights of the entire plant shoot), T represents the average biomass of whole plants treated with strain Jdm2, and C represents the average biomass of whole control plants.

### Biocontrol efficacy of the Jdm2 strain on M. incognita in field tests

The Jdm2 strain was selected for field tests, based on results from greenhouse tests in Shang-qiu, Henan province, in 2012, where tomato cultivar ‘Shanghai Cooperation 903’ was planted. The fields had been continuously cultivated with tomatoes for 8 years prior to the field test, and the incidence of *M. incognita* in these fields has been over 50% since 2008. Each field test consisted of one treatment (with bacterial suspensions of Jdm2) and two controls with four replicates each. The experimental plots were arranged randomly. Each plot was 84 m^2^ with 150 tomato seedlings. In the treatment group, each seedling was inoculated with 200 mL of antagonist suspension (1.7×10^6^ CFU/mL) upon transplantation. In control 1, each seedling was inoculated with an equal volume of water instead of an antagonist suspension. In control 2, each tomato seedling was treated with 100 mL of 9 ppm avermectins. The avermectins, streptomycete-derived macrocyclic lactones originally isolated as antiparasitic agents, have also demonstrated high potencies evaluations against the *Meloidogyne* spp. [35]. The disease severity was recorded on 15, 30 and 60 day post-transplantation, and the plant heights in each treatment were calculated at the same time.

### DNA extraction and PCR amplification (16S rRNA)

One composite rhizosphere soil sample taken per plot consisted of roots of 10 randomly selected tomato plants. Plants and soil were removed from the pots, only the soil adhering to the roots was considered as rhizosphere soil, and the rhizosphere soil was collected by shaking off from the roots in air. After sieving (2 mm), each composite of fresh soil samples were immediately transferred to the laboratory and stored at -70°C.

Total soil DNA was extracted by placing approximately 500 mg of soil in tubes containing lysing matrix and then using the Fast DNA™ SPIN Kit for Soil (Qbiogene, Carlsbad, CA, USA) following the manufacturer's protocol. Extracted DNA was amplified and used as a template with primer pair F984GC-R1378 to obtain fragment 968-1401 [36], suitable for gradient gel analysis. In order to amplify the 433 bp fragment, the reaction mixture was as follows: 2.5 µL of 10× *Taq* buffer, 3.75 µL of MgCl_2_ (25 mmol/L), 2 µL of dNTP mix (2.5 mmol/L each), 0.25 µL of forward and reverse primers (each 10 µmol/L), 1 µL of template DNA (50–100 ng), and 0.5 µL of *Taq* DNA polymerase (5 U/µL; (Takara Intl., Japan). For amplification of environmental DNA, 1 µL of bovine serum albumin (5 mg/mL) was used to prevent inhibition of PCR [37]. Sterilized distilled water was added to a final volume of 25 µL. PCR conditions were as follows: initial denaturation at 94°C for 5 min, amplification over 35 cycles of 1 min denaturation at 94°C, 1 min primer annealing at 60°C, and 2 min primer extension at 72°C, followed by a final step of 10 cycles of 0.5 min at 60°C and 1 min at 72°C, and then cooling the mixture to 4°C. Resulting PCR products were first analyzed by electrophoresis on 1.5% (wt/vol) agarose gels with ethidium bromide staining [38].

### DGGE of PCR products

Amplicons from PCR were analyzed by DGGE using a DCode system (Bio-Rad, Hercules, CA, USA) on 8% polyacrylamide gels (37.5:1 acrylamide-bisacrylamide) in 0.5× TAE (20 mmol/L Tris-acetate, 0.5 mmol/L EDTA) buffer. For the separation of amplicons, 35 to 55% denaturing gradients were used. A 100% denaturing solution was defined as 7 mol/L urea and 40% formamide. Electrophoresis was initiated by pre-running at a voltage of 200 V for 10 min and then at a constant voltage of 80 V for 13 h at 60°C. The DGGE gels were silver-stained, according to the method of Sanguinetti et al. [39]. Gels were analyzed using Molecular Analyst 1.61 software (Bio-Rad) to acquire relative signal intensities of detected bands. Dendrograms were constructed based on pairwise Pearson correlation indices by means of unweighted pair group method using arithmetic averages (UPGMA). The pairwise Pearson correlation indices were used to test for significant difference of community compositions between treatments [40].

### Effect of Jdm2 inoculation on bacterial communities in tomato rhizospheres

For cultivation-independent analysis, DNA extraction with the method mentioned above was used. A nested PCR approach was employed for specific amplification of *Pseudomonas* spp. and *Bacillus* spp. 16S rRNA gene fragments. The first taxon-specific PCR amplification for *Pseudomonas* spp. was carried out using the primer pair F311/R1459 [41]. PCR products were diluted 1:10 and used as a template for the second PCR with bacterial primers F984GC and R1378, with subsequent separation by denaturing gradient gel electrophoresis (DGGE). For the *Bacillus*-specific amplification, the primer pair BacF/R1378 was used as described previously [42]. The DGGE method was conducted according to the method described above on the BIORAD DCode system.

### Identification of the antagonist

The morphological and physiological characteristics of the Jdm2 strain were identified according to Bergey's Manual of Determinative Bacteriology.

The genomic DNA of the strain Jdm2 used in the greenhouse tests was extracted. The 16S rRNA gene of the antagonist was sequenced and subjected to a Blast search in the NCBI Nucleotide Sequence Database [43], Accession number: KF001839.

### Data analysis

The analyses of the variances of biocontrol efficacy and biomass of tomato were performed using the SAS general linear model (GLM) procedure (SAS Institute, Version 9.2, Cary, NC, USA). The mean comparisons were conducted using one-way analysis of variance (ANOVA) and the Tukey-Kramer multiple comparison tests (*p*<0.05).

## Supporting Information

Figure S1 **Denaturing gradient gels with fingerprints of bacterial communities from tomato rhizosphere soils.** The fingerprints of bacterial communities were generated by separation of 16S rRNA gene fragments. ‘‘A’’ was on behalf of the controls amended with Avermectins, ‘‘B’’ was on behalf of the controls amended with Jdm2, and ‘‘C’’ was on behalf of the treatments amended with water. The number (0, 15, 30, 60) following the abbreviation letters ‘‘A’’, ‘‘B’’ and ‘‘C’’ represented the sampling day after inoculation. The same as Figure S2.
(TIF)

Figure S2 ***Bacillus*** **-specific (A) and** ***Pseudomonas*** **-specific (B) DGGE fingerprints corresponding UPGMA clusters for soils amended with Jdm2 or Avermectins or not.**
(TIF)
