# Supplementary figures and images for: Isolation and Characterization of a Rhizobacterial Antagonist of Root-Knot Nematodes
Source: PLoS One. 2014 Jan 21;9(1):e85988. doi: 10.1371/journal.pone.0085988 (PMC3897607; doi:10.1371/journal.pone.0085988)

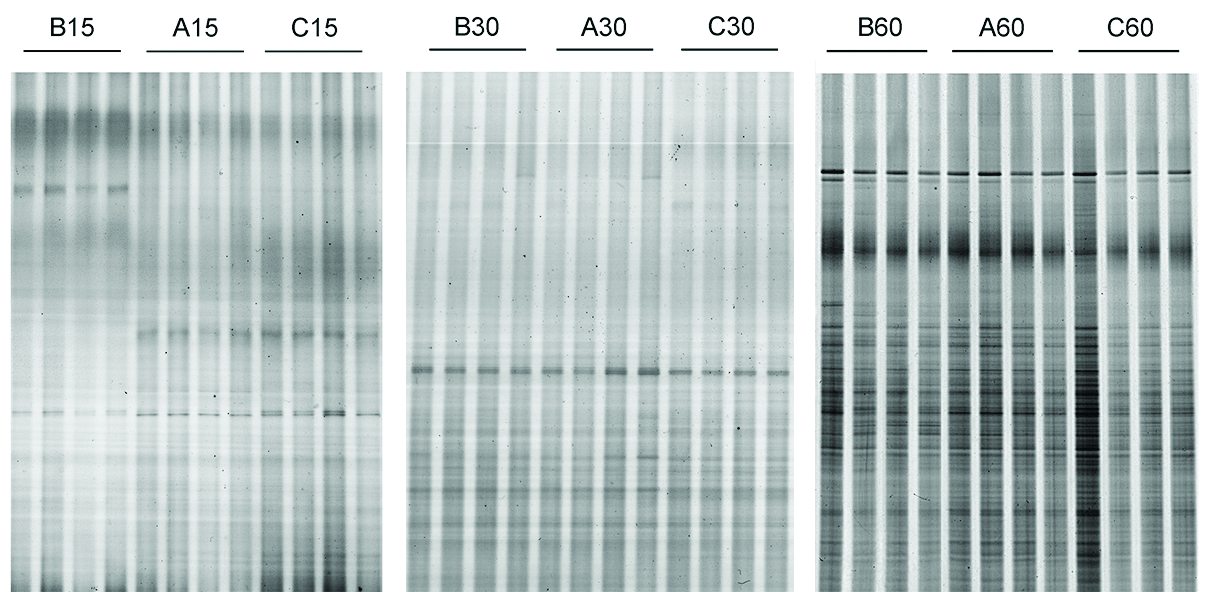

Supplement: Figure S1 — Denaturing gradient gels with fingerprints of bacterial communities from tomato rhizosphere soils. The fingerprints of bacterial communities were generated by separation of 16S rRNA gene fragments. ‘‘A’’ was on behalf of the controls amended with Avermectins, ‘‘B’’ was on behalf of the controls amended with Jdm2, and ‘‘C’’ was on behalf of the treatments amended with water. The number (0, 15, 30, 60) following the abbreviation letters ‘‘A’’, ‘‘B’’ and ‘‘C’’ represented the sampling day after inoculation. The same as Figure S2. (TIF) [file pone.0085988.s001.tif]

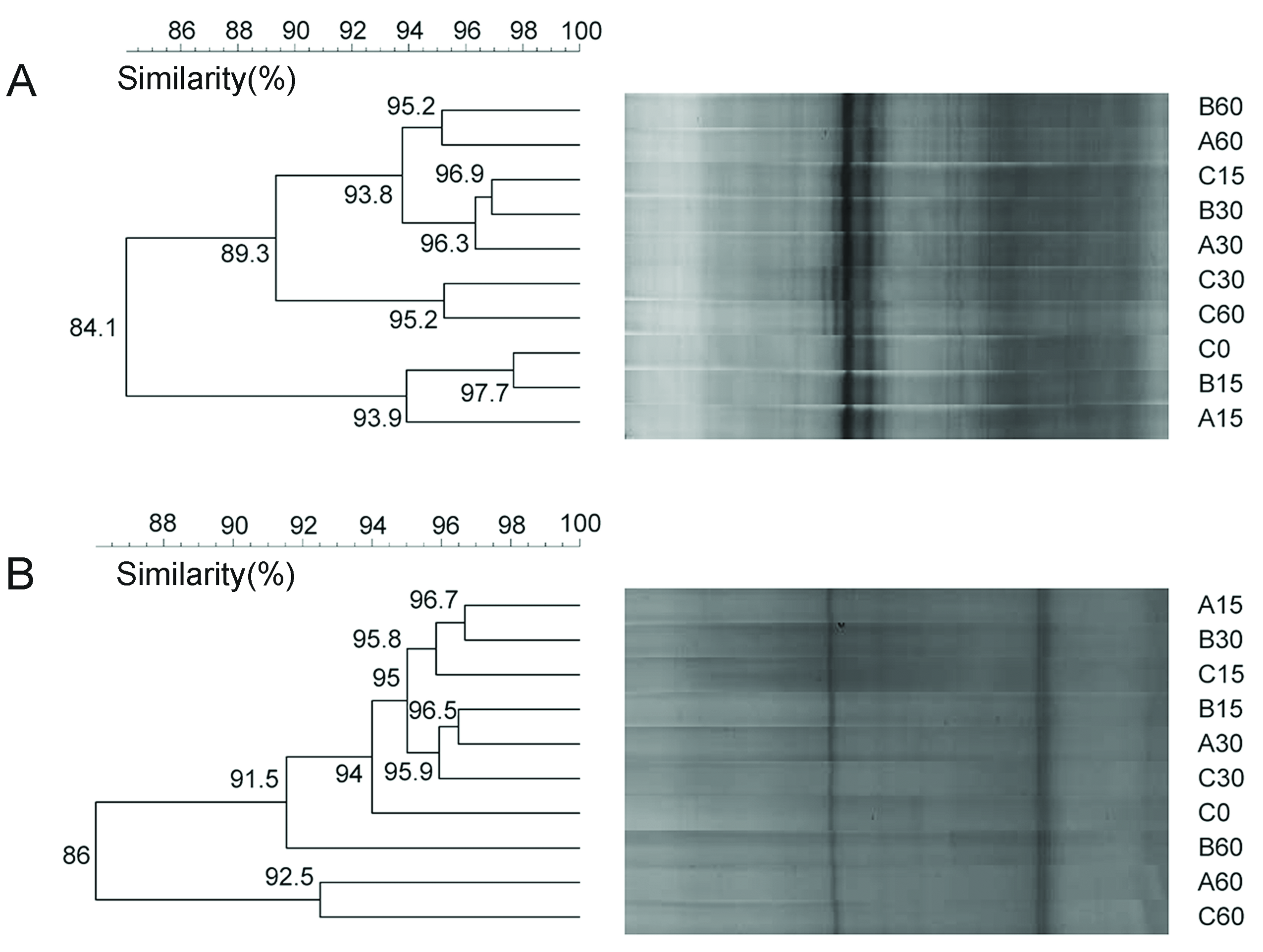

Supplement: Figure S2 — Bacillus -specific (A) and Pseudomonas -specific (B) DGGE fingerprints corresponding UPGMA clusters for soils amended with Jdm2 or Avermectins or not. (TIF) [file pone.0085988.s002.tif]
